# Quality and effectiveness of different approaches to primary care delivery in Brazil

**DOI:** 10.1186/1472-6963-6-156

**Published:** 2006-12-05

**Authors:** Erno Harzheim, Bruce B Duncan, Airton T Stein, Carlo RH Cunha, Marcelo R Goncalves, Thiago G Trindade, Mônica MC Oliveira, Maria Eugênia B Pinto

**Affiliations:** 1 Departamento de Medicina Social da Faculdade de Medicina da Universidade Federal do Rio Grande do Sul (UFRGS), Brazil; 2 Fundação Faculdade Federal de Ciências Médicas de Porto Alegre (FFFCMPA), Brazil

## Abstract

**Background:**

Since 1994, Brazil has developed a primary care system based on multidisciplinary teams which include not only a physician and a nurse, but also 4–6 lay community health workers. This system now consists of 26,650 teams, covering 46% of the Brazilian population. Yet relatively few investigations have examined its effectiveness, especially in contrast with that of the traditional multi-specialty physician team approach it is replacing, or that of other existing family medicine approaches placing less emphasis on lay community health workers.

Primary health care can be defined through its domains of access to first contact, continuity, coordination, comprehensiveness, community orientation and family orientation. These attributes can be ascertained via instruments such as the Primary Care Assessment Tool (PCATool), and correlated with the effectiveness of care. The objectives of our study are to validate the adult version of this instrument in Portuguese, identify the extent (quality) of primary care present in different models of primary care services, and correlate this extent with measures of process and outcomes in patients with diabetes, hypertension and coronary heart disease (CHD).

**Methods/Design:**

We are conducting a population-based cross-sectional study of primary care in the municipality of Porto Alegre. We will interview a random sample totaling 3000 adults residing in geographic areas covered by four distinct models of primary care of the Brazilian national health system or, alternatively, by one nationally prominent complementary health care service, as well as the physicians and nurses of the health teams of these services. Interviews query perceived quality of care (PCATool-Adult Version), patient satisfaction, and process indicators of management of diabetes, hypertension and known CHD. We are measuring blood pressure, anthropometrics and, in adults with known diabetes, glycated hemoglobin.

**Discussion:**

We hope to contribute not only by validating the PCATool-Adult Version for use in Brazil, but also by furnishing ample data concerning the appropriate mix of health care professionals in the primary care team, a question of international import. Once validated, future use of this instrument should help direct advances aiming at improving the quality of primary care in Brazil.

## Background

With the declaration of Alma-Ata, the World Health Organization (WHO) pronounced that health systems emphasizing a strong and effective primary care are the best approach to achieving "Health for All"[1]. In the decades that have followed, many countries have worked to direct their health systems toward the model envisioned at Alma-Ata. Brazil joined this effort more recently, defining as the basis of its primary care system the so-called Programa Saúde da Família (PSF or Family Health Program), a multidisciplinary team composed of a physician, a nurse, two nurse's assistants, and 4–6 lay community health workers delivering care to a geographically defined population. More recently, a dentist and dental assistant have been added to the team. The number of teams has grown, from their start in 1994, to a current total of 26,650, and cover 46% of the Brazilian population [2-8]. As such, the PSF represents the nearest approximation to the primary care model envisioned at Alma-Ata that is currently implemented by a major developing country, and may be an example to be emulated by other such countries.

Yet important doubts remain in Brazil as to whether this multidisciplinary team model is the most appropriate one, and to what extent it is responsible for improvement in health indicators such as an increased coverage of adequate prenatal care and deceased infant and stroke mortality, seen in Brazil [9-11] over the past decade and a half.

In Porto Alegre, capital of Brazil's southernmost state, other family physician models of primary health care preceded the advent of the PSF, and the primary care health network includes institutions – the Centro de Saúde Escola Murialdo (Murialdo) and the Serviço de Saúde Comunitária of the Hospital Nossa Senhora da Conceição (Conceição) – that maintain these models of care. In these services, primary care is based on family physicians and delivered to geographically defined communities, but with a lesser emphasis on community health workers. In addition, more traditional teams of physician specialists provide primary care with lesser outreach activities and community participation at the traditional Unidades Básicas de Saúde (UBS), or Basic Health Units, now being phased out across the country. Outside of the public sector, following a national trend of reorganization of primary care services of complementary health care systems, a prominent health care provider, the Caixa de Assistência dos Funcionários do Banco do Brasil (CASSI) implemented in 2004 a system based on family physicians but lacking a geographically-defined population and, given its occupational base, presenting additional peculiarities. Given the major shift in Brazil's primary care model, this diversity of services with different organizational features in Porto Alegre offers a unique opportunity to compare the processes and the results obtained in the different primary care models in order to guide further development of public and private primary care services in Brazil and elsewhere.

Primary health care can be defined through the domains (or attributes) of access to first contact, longitudinality, coordination, comprehensiveness, community orientation, and family orientation [12]. These attributes, recognized as the structural bases of the primary care process, are associated with quality of services [13], patient satisfaction[14] and health system effectiveness[15], efficiency [16] and equity[17]. However, Blumenthal[18], in a review of the effectiveness of primary care to vulnerable population groups, concluded that the evidence base required strengthening, and that further investigations that clearly define the attributes being evaluated and their association with effectiveness are necessary. Evidence of this nature is also scarce in Brazil. Thus, the generation of additional knowledge linking primary care models and process attributes with health outcomes is vitally important.

Starfield et al. have developed an instrument capable of measuring the presence and extent of the attributes in question[19], the Primary Care Assessment Tool (PCATool). This tool provides scores of the cited attributes as well as a general score of the quality of the process of primary care. Thus, its use in evaluating the quality of offered care, through these attributes, permits the evaluation of the degree of success of primary care services in achieving excellence in their practices. It is applied in two complementary versions, one to primary health care services users and the other to primary care providers.

The disease burden in Brazil results primarily from diseases in adults[20]. In adults, the principal causes of morbidity and mortality are non-communicable, especially cardiovascular, diseases. As such, hypertension [21] and diabetes [22] are important foci of care, and the prevention and management of these diseases [21-25] through programmatic actions have become priorities in both the PSF and the other primary cares services under study[26]. Besides this, patients who present known coronary heart disease (CHD) require special management in primary care in order to avert new events. The secondary prevention of cardiovascular events in such patients is strongly evidence-based and of easy implementation in primary care [27], quality indicators having already been developed for its evaluation[28].

The definition of indicators of the quality of process – scores of primary care attributes – and the investigation of their correlation with intermediate outcomes of patients with hypertension, diabetes and known CHD are important to evaluation of the impact of primary care actions. Such population-based inquiries linking primary care service models with quality of care, patient satisfaction and health outcomes are fundamental in the defining the direction of future public health care actions.

## Objectives

### Main Objective

To evaluate, via the PCATool-Adult Version, the quality of care offered to adults through different models of care currently present in the primary care network of the public (PSF, UBS, Conceição and Murialdo) and complementary (CASSI) health care systems in Porto Alegre, and to compare this perceived quality of care with process measures and outcomes in patients with hypertension, diabetes mellitus and known CHD.

### Specific Objectives

1. To validate the Brazilian version of the PCATool-Adult Version;

2. Utilizing this PCATool, to characterize and compare the quality of adult primary care offered by different primary care models in the network of public services and in one complementary health service (CASSI) ;

3. To investigate the association of the extension of these attributes with

a. patient satisfaction,

b. degree of self-perceived health,

c. blood pressure control in hypertension,

d. glycemic control in diabetes,

e. adequate management of patients with known CHD,

f. frequency of hospitalization in the past year.

4. To compare levels of these same outcomes across the different models of care.

## Methods/Design

### Study Design, Setting and Participants

We are conducting a population-based cross-sectional study of four important models of primary care services within the Brazilian national health system, as well as a clinically based cross-sectional study of the primary care service of a prominent national complementary health system, in the city of Porto Alegre. Field work started in June, 2006, and data collection is scheduled to end in May, 2007.

We are studying both covered adults and care providers. Covered adults are defined, for the public services, as all individuals over 18 years of age residing in Porto Alegre, and for the complementary service as adults covered by family medicine teams of the CASSI in Porto Alegre. The care providers are those physicians and nurses who provide the primary care services offered through the PSF, UBS, Murialdo or Conceição, or in the health teams of the CASSI, in Porto Alegre.

Adults who are deemed by the field coordinator as unable to answer questionnaires, who are seen primarily by health services in another city, who have been living in the area for less than one year and who had their last medical visit before 1996 are being excluded.

Cluster sampling of the households will be used to select individuals covered by the public systems. In the selection of individuals covered by the UBS units and PSF teams, the adult population of Porto Alegre was initially divided into strata defined by the municipality's health districts. Next, we randomly selected half of the UBS units and PSF teams of each municipal health district for investigation. For Conceição and Murialdo, all of the health units (12 and 7, respectively) were selected. Next, within each of these units or teams, we are randomly selecting census tracts. Within each selected tract, we are systematically selecting households using a random start, and interviewing all household members (to a maximum of 40 per tract) who fulfill selection criteria. The number of individuals interviewed in relation to each unit/team will be proportional to the population covered by that unit/team within the service.

The CASSI sample is being randomly selected using the list of families of patients over 18 years of age covered by the Porto Alegre Family Health Teams. All covered household members of families selected are interviewed.

All physicians and nurses who belong to the teams selected for the study will be interviewed.

### Power Calculations

Sample size was calculated using the EPI-INFO 6.0 statistical package[29] to describe and compare proportions, using data from an earlier study we conducted to validate the pediatric version of PCATool [30] and data from the national literature on the prevalence, recognition and control of hypertension[31] and diabetes[32].

Our study received funding at two moments. The first funding was to support the objective of evaluating the quality of health care in PSF teams only. At this point, we calculated sample size necessary to evaluate users and professionals of this model of primary health care. To fulfill the objective of validating the PCATool-Adult Version, we estimated that 400 people would be needed, based on the necessity of 5 respondents for each item of the 80 item questionnaire[33]. For descriptive analyses of the quality of care in the PSF, we estimated that a sample size of between 653 and 1280 was needed to generate a 95% confidence level with precision of 3%, assuming a design factor of 1.2 to correct for the effect of cluster sampling, and estimating that the proportion of users with a high PCATool primary care attributes score varied from as high as 50% to as low as 15%. Estimating that approximately 10% of those initially identified would refuse or not be at home during at least 3 visits on different days and at different times, we added 120 individuals to the sample to be initially procured to evaluate the PSF, producing a total of 1400 adults. Additionally, to the extent that we interview individuals who report using complementary health systems rather than the PSF, the sample will be increased accordingly so as to achieve our target.

At a second moment, we obtained further funding from an additional institution, permitting the expansion of our objectives to include the evaluation of the additional primary care models of the SUS (Murialdo, SSC, and UBS) and CASSI. The minimum sample calculated as necessary for each of these other primary care models, to allow a comparison of perceived quality of care by its users vs. that perceived by those of the PSF, was 300 users. This calculation was based on an alpha error of 5%, 80% statistical power, a difference of at least a 12% in the proportion of users with a high overall primary care attribute score, and the proportion of users with a high score varying from 15 to 50% in the health service model of lowest quality. For the same reasons as exposed with respect to the final PSF sample, we opted to increase the number of adults to be initially procured to 400 for each of these additional services. Similarly, additional interviews are being performed to the extent that users of complementary health services are identified in the coverage areas of the public health service samples. Thus, our total sample is 3000 users (1400 for the PSF, and 400 each for Murialdo, SSC, UBS and CASSI).

We assume that approximately 1/3 of hypertensive subjects will have good blood pressure control (arterial blood pressure < 140/90 mmHg) and approximately 1/3 of those having diabetes will be in good metabolic control (glycated hemoglobin < 7, 0%) [34]. For an alpha error of 5%, statistical power of 80%, assuming 50% of patients with diabetes or hypertension with a high overall primary care quality score have good control vs. only 25% of those with a low score, we would need a sample of 130 diabetic and 130 hypertensive patients. Assuming that we will identify 30% of the sampled population as having hypertension and 5% as having diabetes, we should identify 900 as hypertensive and 150 as having diabetes, respectively. If recruitment of diabetes is inadequate, sampling following the same approach described above will be extended to enroll additional patients with diabetes who will be considered only for the hypotheses related to this disease.

### Ethical Issues

This project was approved by the Committee on Ethics in Research of the Federal University of Rio Grande do Sul and by similar committees governing research in the health care services under study. The project is being discussed with district level health administrators, with the health teams which will be evaluated and with the population through the local health councils of the target communities. Only after completing this discussion with involved parties have we initiated data collection in each community. Written informed consent is being obtained from all participants. All individuals identified for the first time as probable cases of diabetes and hypertension, as well as those with hypertension and/or diabetes whose examination suggests poor control are being referred to their point of care for evaluation and follow-up.

### Measurements

We are obtaining data from the covered population through structured interviews, composed of instruments measuring three distinct sets of variables:

- social-demographic characteristics, cardiovascular risk factors (sedentary lifestyle, smoking, known hypercholesterolemia, alcohol consumption, dietary patterns), known hypertension, diabetes and CHD, process outcomes related to these diseases (receipt of exercise, nutrition and anti-smoking counseling; use of aspirin, metformin and diuretic thiazide use, among others) and number and causes of hospitalizations in the last year;

- user satisfaction[35] (The chosen instrument, previously validated in Brazil, is made up of 12 questions about the different aspects of health care – access, kindness, trust, physician performance, guidance, visit scheduling, and an overall evaluation – with Likert-type answers ranging from 1 to 5 represented by drawings of faces showing 5 different expressions of satisfaction); and

- the presence and the extent of the 4 essential primary care attributes (access, continuity, coordination and comprehensiveness) and of the derived attributes (family orientation and community orientation), as well as the degree of affiliation to the health service (PCATool-Adult Version[19]).

We also are measuring weight, height, and waist and hip circumferences. We are measuring blood pressure twice during the household visit, with a time interval of at least 3 minutes between the 1st and the 2nd measurements, following to the guidelines of the World Health Organization[36]. We measure glycated hemoglobin in participants with known diabetes, scheduling blood collection in the health units by a nurse assistant from a contracted laboratory. Blood samples are processed through high performance liquid chromatography (HPLC – Bio-Rad), according to the American Diabetes Association standard[37].

### Logistical Aspects

Having completed training and certification, nine study coordinators and a team of 20 interviewers are currently in the field collecting data and, when indicated, scheduling blood collection, in accordance with the guidelines of the study's manual of operations. Parts of interviews and measurements (weight, height, hip and waist circumference) of approximately 10% of the sample are being repeated in order to ensure the quality of data collection and to permit the validation of the PCATool. The physicians and nurses of the selected health units are interviewed by field coordinators in their place of work.

Data entry is performed by scanning the questionnaires and later converting the images to an SPSS 13.0 database, using *Teleform*^® ^software (Cardiff, Vista, California) and its sub-modules *reader *and *verify*. Checking for errors in the database is performed upon data entry.

### Data Analysis

#### Validation of the PCATool-Adult Version

The process of validation involves the following: translation, reverse translation, debriefing, content and construct validity, internal consistency and reliability (precision and stability over time), as well as additional steps described below associated with Likert-type scale questions[38,39].

After translation from English into Portuguese by a native Portuguese speaker, and reverse translation back into English by a native English speaker, the original and reverse translation versions were compared, and mistakes corrected. As the original instrument was developed for self-administration; we converted it into an interviewer-administered questionnaire. Additional adaptations were necessary to take into account the cultural characteristics of the population and of the Brazilian national health services.

Next, debriefing consisted of the application of the questionnaire to 6 individuals similar to the population under study, at which point the degree of understanding of each of the questions was assessed until there were no further doubts. The original instrument has already been validated for content in the United States, a process in which its content validity was defined through expert opinion [30]. Thus, content validation for its use in Brazil consisted in verification, through debriefing by those interviewed, of that their comprehension of the content was consistent with the intended theoretical content. This procedure was also used for adapting the language used. During these steps, some questions were excluded and others modified.

Construct validation refers to the redefinition of which items (questions) of the PCATool-Adult Version will be used to compose each of the attributes to be ascertained. This definition is produced via factorial analysis, through selection of factors presenting three or more items (questions) having loadings of greater than 0.35 without additional loadings of larger size on other factors. This analysis is done extracting principal components using the VARIMAX option. Once the analysis is performed, we evaluate which of the identified factors relate to the theoretical concepts of the primary care attributes, thus identifying a factor to represent each of these attributes.

As previously mentioned,10% of the interviews are repeated over a 1–3 months interval after the first interview to define precision and stability over time. We will calculate the scores for each attribute obtained in the two distinct moments and compare them using the Wilcoxon and Kappa test.

In terms of internal consistency, each primary care attribute defined through factorial and conceptual analysis should have a Cronbach's α > 0.70. After final construction of these attributes, 5 criteria are used to verify that meet the assumptions of the Likert scale: item-convergent validity, through an item-total correlation > 0.30; item-discriminant correlation, through the "scale success ratio", that is, the correlation of each item with its identified attribute should be greater than the correlation of this item with other attributes; intraclass correlation of the items of each attribute; interval of the correlation of the items of each attribute; and reliability of the scores (Cronbach's α). This instrument validation process will be performed in conjunction with the author of the original instrument (B. Starfield) [38-40].

Descriptive statistical analyses (sample characteristics, evaluation of health services, user satisfaction, self-rated health status, and the extension of the primary care attributes present) will take into account the sampling strategy (clusters), using the STATA 8 [41] statistical software. These data will be presented using their means and standard deviations, confidence intervals and proportions. Estimate of the extension of these attributes in the conjunct of all public primary care services in Porto Alegre will be produced weighting original responses so as to reflect the fraction of the population covered by each type of service.

Hypothesis testing, for example that a greater extension of primary care attributes associates with greater control of blood pressure, will be performed using continuous and categorical expressions of outcomes. We will compare means between public services and between these and the private service through Student's "t" test and one-way analysis of variance (ANOVA), and proportions through chi-square testing. We will employ a 5% two-tailed confidence level for all statistical tests. In order to adjust for possible confounders in the association of the primary care attribute scores with categorical measures of health status, satisfaction, and level of control of diabetes and hypertension, we will construct logistic regression or log-binomial models.

## Discussion

Documentation of the quality of different primary care models in Brazil and its association with patient satisfaction and disease outcomes is important not only to guide the continuing local and national implementation of the primary care system but also to orient the planning of primary care systems in other developing countries. We trust that this study will contribute not only by validating an internationally recognized instrument – the PCATool-Adult Version for use in Brazil, but also by furnishing ample data for debate as to the appropriate mix of health care professionals in the primary care team.

The process of validating the PCATool is a complex and sophisticated one. It is worth noting that the important associations of health outcomes with the extent of primary care as assessed with this instrument have been reported from studies using adequately validated versions.

The PCATool-Adult Version, once validated, will find use in evaluation of adult health services, public and private, in other Brazilian settings, permitting evaluations based on the experience of the users and professionals of these systems. As such, it should widen the options available to guide improvement of health care delivery in Brazil. Its future use will permit comparisons between different models of care and between similar models in different settings. Tracking of temporal trends in the quality of care within a given service model will also be possible.

## Competing interests

The author(s) declare that they have no competing interests.

## Authors' contributions

EH, BBD, ATS, CRHC, MO and MEB participated in the conception and design of the study. EH, BBD, MRG, and TGT drafted the manuscript. All authors have read, revised and approved the final manuscript.

## Pre-publication history

The pre-publication history for this paper can be accessed here:


